# Evidence of mycobacterial disease in COPD patients with lung volume reduction surgery; the importance of histological assessment of specimens: a cohort study

**DOI:** 10.1186/1471-2466-14-124

**Published:** 2014-08-02

**Authors:** Anjali Char, Nick S Hopkinson, David M Hansell, Andrew G Nicholson, Emily C Shaw, Samuel J Clark, Philip Sedgwick, Robert Wilson, Simon Jordan, Michael R Loebinger

**Affiliations:** 1 St. George’s, University of London, London, UK; 2 Royal Brompton and Harefield NHS Foundation Trust, London, UK; 3 National Heart and Lung institute, Imperial College, London, UK

**Keywords:** COPD, Mycobacterium, Non tuberculous mycobacterium (NTM), Lung volume reduction surgery (LVRS)

## Abstract

**Background:**

Patients with COPD are at risk of non-tuberculous mycobacterial infection (NTM). This study examined the histology of lung tissue from COPD patients following lung volume reduction with particular focus on evidence of mycobacterial infection.

**Methods:**

Retrospective histological study of 142 consecutive lung volume reduction surgical specimens (126 separate patients) at Royal Brompton Hospital between 2000 – 2013, with prospectively collected preoperative data on exacerbation rate, lung function and body mass index.

**Results:**

92% of patients had at least one other histological diagnosis in addition to emphysema. 10% of specimens had histological evidence of mycobacterial infection, one with co-existent aspergilloma. Mycobacteria were only identified in those patients with granulomas that were necrotising. These patients had higher exacerbation rates, lower TLCO and FEV_1_.

**Conclusion:**

A proportion of severe COPD patients will have evidence of mycobacterial infection despite lack of clinical and radiological suspicion. This may have implications for long-term management of these patients.

## Background

Non-tuberculosis mycobacteria (NTM) are a group of widely distributed environmental mycobacteria (other than tuberculosis and leprosy). There are over 100 species with varying degrees of pathogenicity [1]. Pulmonary disease is the most common manifestation and is commonly diagnosed using American Thoracic Society (ATS) guidelines. A diagnosis requires pulmonary symptoms, and nodular or cavitatory opacities on imaging, in addition to positive cultures from two or more separate sputum samples or one positive culture from tissue/bronchial lavage samples [2,3]. The prevalence of NTM infection is increasing worldwide which may be due in part to increased exposure and numbers of susceptible patients [4].

NTM are opportunistic bacteria and are therefore more likely to cause disease when there are defects in local or systemic host immunity. Patients with pre-existing lung conditions, such as COPD, cystic fibrosis (CF) and bronchiectasis, are shown to have a higher association with NTM infection [2,5,6].

Lung volume reduction surgery (LVRS) and bullectomy are surgical procedures directed at COPD patients in whom optimal medical treatment has failed to reduce their symptoms. These procedures improve lung mechanics in heterogeneous emphysema by removing the areas of lung with the greatest destruction [7-10]. These procedures provide a resected tissue specimen, which is routinely sent for histopathological analysis. We have previously reported the safety outcomes in this population [11]. This study assessed the histology reports of patients undergoing LVRS and bullectomy for histological diagnoses in addition to emphysema, with a particular reference to evidence of mycobacterial infection.

## Methods

The clinical records of 126 consecutive patients who underwent LVRS or bullectomy at the Royal Brompton Hospital from 2000–2013, were retrospectively reviewed for demographic, clinical, lung function, surgical, radiological, microbiological and histological data. All patients underwent pulmonary rehabilitation prior to surgery and had a thorough pre-operative work up, including chest radiographs (CXR) and/or CT scans and lung function tests. Indications for LVRS/bullectomy: emphysema with destruction and marked hyperinflation; substantial bulla identified on CXR; a marked functional impairment; a marked reduction in activities of daily living; a failure of maximum medical treatment to reduce symptoms; heterogeneous disease with obvious apical target areas [7].

The LVRS or bullectomy was performed unilaterally using lateral thoracotomy or bilaterally via median sternotomy. Video assisted thoroscopic surgery (VATS) procedure was used for either bilateral or unilateral procedures.

Pathological diagnoses in addition to emphysema mentioned in histology reports were noted, with special focus on evidence of mycobacterial infection (necrotising granulomas with or without positive Ziehl-Neilson (ZN) staining for acid-fast bacilli (AFB)). All lung nodules identified pre-operatively by imaging or identified in resected specimens were recorded. Exacerbation rates were defined as the number of COPD exacerbations for the year prior to surgery as recorded in the clinical records.

Statistics are reported as mean ± standard deviation (SD). The groups (evidence of mycobacterium or not) were compared in the following variables: sex, age, BMI, exacerbation rate, lung function pre-operation (% predicted), pre-operation FVC (% predicted), pre-operation TICO (% predicted), pre-operation TLC (% predicted) and pre-operation RV (% predicted). Groups were compared in the distribution of sex using Fisher’s Exact test since the Chi-Squared test was invalid. If the distributional assumption of normality could be made, groups were compared using the independent samples t-test (test statistic denoted by t). If equal variances could not be assumed between groups, Satterthwaite’s approximation to the degrees of freedom was made. The Mann–Whitney test was used to compare the mycobacterium groups in exacerbation rate since the assumption of normality could not be made. A multivariate analysis was performed using binary logistic regression, with mycobacterium status (present versus absent) as the outcome variable. All explanatory variables described above were entered into the model. The explanatory variables were categorised as follows: sex (male, female), age (<60, ≥60 years), BMI (<18.5 kgm^−2^, ≥18.5 kgm^−2^), exacerbation rate (<2 per year, ≥2 per year), FEV1 (% predicted) (<40%, ≥40%), FVC (% predicted) (<80%, ≥80%), TICO (% predicted) (<40%, ≥40%), TLC (% predicted) (<125%, ≥125%) and RV (% predicted) (<200%, ≥200%). The odds ratios were adjusted for all other explanatory variables in the model. The critical level of significance was set at 0.05 (i.e. 5%). 95% Confidence Intervals are denoted by 95% CI. The Research Office of the Royal Brompton has ruled that this is a service evaluation and that formal ethical approval was not required.

## Results

The demographic and surgical data of the 126 patients are shown in Table 1. In total, 142 specimens were obtained from 142 procedures, including 16 patients who had two operations on separate occasions. The mean age was 57 years and 75% of the patients were male. The majority (95%) were Caucasian, with 5 Asian and 1 African patient. Pulmonary function tests showed a severe obstructive lung disease with a mean FEV_1_ of 36.3% of normal, and severe air trapping with a residual volume of >200% of normal. There was an average of 2.59 exacerbations per year. Early mortality (≤30 postoperative days) was 1 of 126 patients. In view of the severity of the emphysema over 96% patients were on inhaled steroids. There was some missing data: 12 patients had no exacerbation rate recorded in clinical notes; 3 patients had no pre-operative FEV_1_ and FVC recorded; 5 patients had no pre-operative TLCO; 6 patients had no pre-operative TLC and 7 patients had no pre-operative RV recorded.

**Table 1:** Demographic and surgical data

***Demographic and surgical data***	
No. of patients	126
Total no. of operations	142
Age, yr (mean ± SD) (Range: 29 – 81 yr)	57.3 ± 9.74
Gender M	107 (75%)
F	35 (25%)
BMI (mean ± SD)	23.52 ± 4.16
Exacerbations/year (mean ± SD)	2.59 ± 2.61
FEV_1_, % predicted (mean ± SD)	36.30 ± 16.83
FVC, % predicted (mean ± SD)	81.27 ± 20.52
TLCO, % predicted (mean ± SD)	40.47 ± 16.99
RV, % predicted (mean ± SD)	216.48 ± 59.29
TLC, % predicted (mean ± SD)	128.61 ± 20.43
LVRS Operations	112 (79%)
Bilateral	27 (24%)
Unilateral*	85 (76%)
Right	52 (61%)
Left	33 (39%)
Bullectomy^	30 (21%)

*15 patients had both R and L LVRS done on separate dates.

^1 patient had both R and L bullectomy on separate dates.

BMI: body mass index; FEV_1_: forced expiratory volume in 1 s; LVRS: lung volume reduction surgery. Data shown as mean ± Standard Deviation (SD).

**Figure 1:**
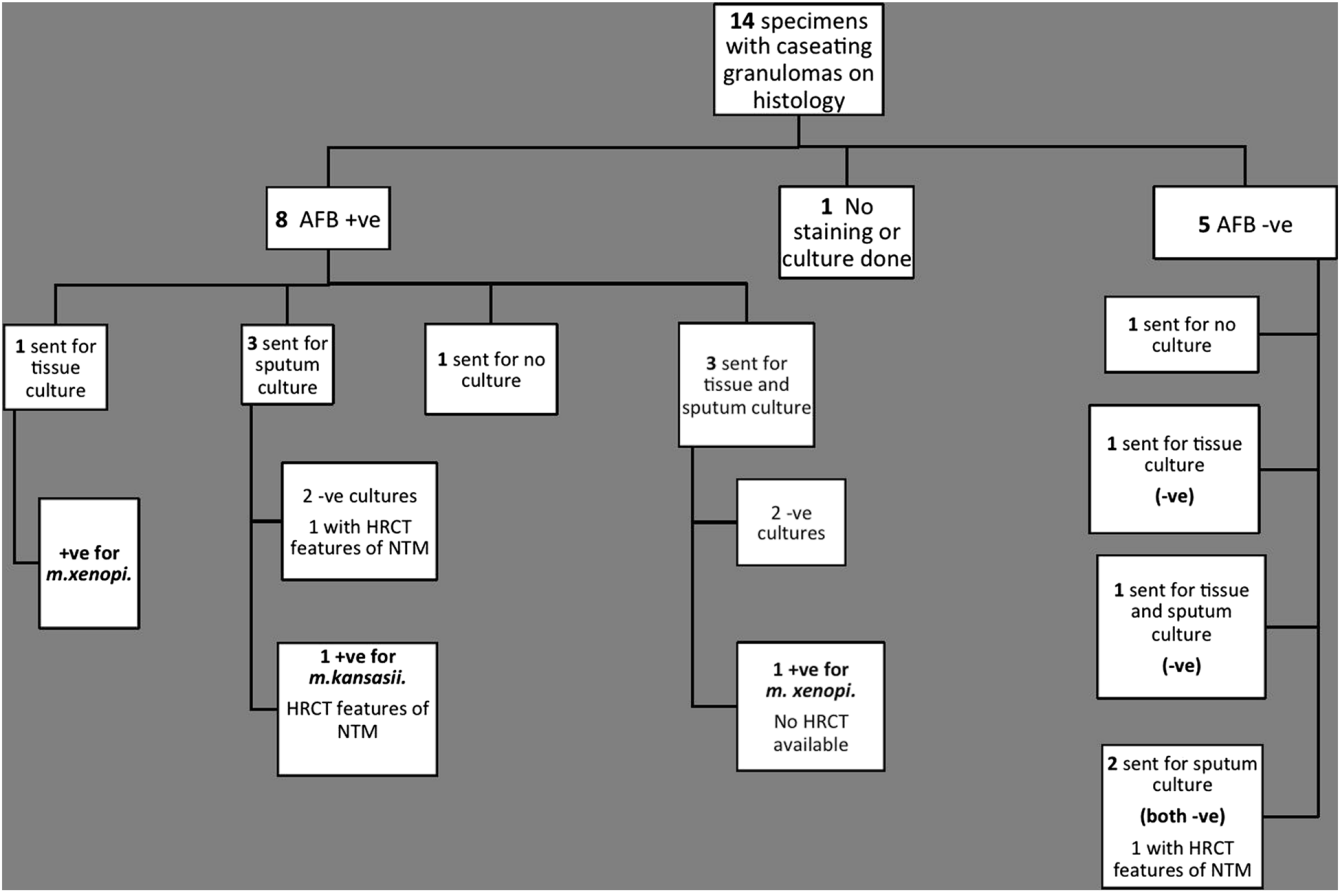
**Flowchart illustrating histopathological results.** 3 of 14 specimens were cultured positive for NTM infection. HRCT: High resolution computed tomography; NTM: Non-tuberculous mycobacterium.

**Figure 2:**
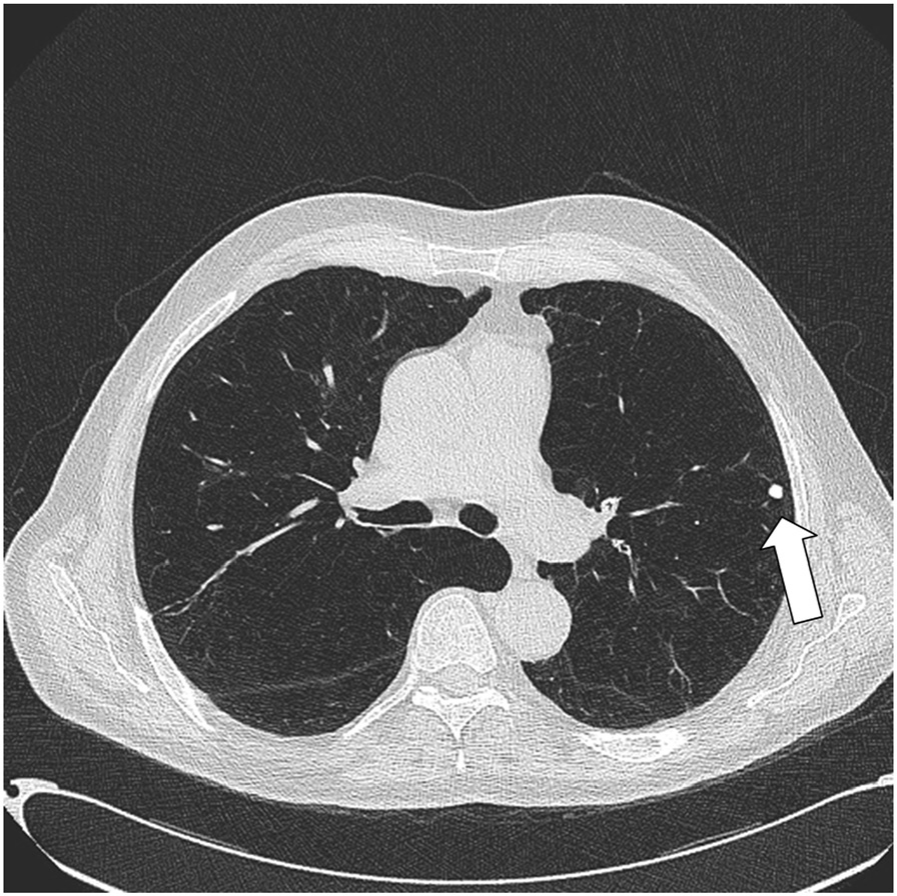
Chest CT scan showing emphysema and nodule (arrow).

Fourteen histological reports had evidence of mycobacterial infection, with one or more necrotising granulomas, and 8 of which showed acid-fast bacilli on Ziehl-Nielsen staining. As there was no clinical suspicion of mycobacterial infection at the time of the operation, a minority (4/14 samples) were sent for mycobacterial tissue culture; and 2 specimens subsequently grew *Mycobacterium xenopi*. Although there were no other tissue cultures, a further 8 patients had sputum samples cultured for mycobacteria with 1 of these samples culturing *mycobacterium kansasii* (Figure 1). In one case in which AFBs were present, an aspergilloma was also noted. Fungal stains on the remaining cases were negative. HRCT scans (median time from operation date 28 days (1–47)) were reviewed retrospectively in the patients with histopathological evidence of necrotising granulomas (13/14 scans available) to specifically look for nodules and CT features compatible with mycobacterial disease. Twelve out of these 13 scans had evidence of small nodules (3-15 mm) (Figure 2). Even with the knowledge of the histological findings, only three scans had features that were suggestive of a radiological diagnosis of non-tuberculous mycobacterial infection.

In addition to evidence of mycobacterial infection, additional histological diagnoses were made in a number of cases as shown in Table 2. There were a further 15 patients with evidence of non-necrotising granulomas. No organisms were seen in the five cases where Ziehl-Nielsen and Grocott staining was undertaken on the granulomatous areas.

**Table 2:** Histological findings other than Emphysema (% of 142 operations, most specimens had more than one)

***Histological findings other than Emphysema (% of 142 operations)***	
Chronic inflammation	54 (38%)
Pleural/Interstitial fibrosis	49 (36%)
Respiratory/Chronic bronchiolitis	28 (20%)
Organising pneumonia	22 (16%)
Reactive mesothelial hyperplasia	17 (12%)
Non– necrotising granuloma	16 (11%)
Nectrotising granuloma	14 (10%)
Hyperplastic type II pneumocytes	7 (6%)
Aspergillus infection	5 (4%)
Malignancies:	3 (2%)
Sarcomatoid carcinoma (not seen on CT)	1
Adenocarcinoma, mod. Differentiated (both seen on CT)	2
Tumourlet	1 (0.8%)
Focal neuroendocrine hyperplasia	1 (0.8%)

A comparison of variables (listed in methods) was made between specimens with evidence of mycobacterial infection (14 of 142) and specimens without (128 of 142). This comparison is shown in Table 3. Patients with histological evidence of mycobacterium infection had a significantly lower pre-operative FEV_1_ (p < 0.001; 95% CI: −17.55 to −7.84) and a significantly higher number of exacerbations/year (p < 0.001) compared to patients without evidence of mycobacterium infection. In addition, patients with histological evidence of mycobacterium infection had a significantly lower TLCO (p < 0.001; CI: −18.05 to −5.85). The independent effect of the variables upon mycobacterium (absent or present) was further investigated using binary logistic regression. Exacerbation rate remained as independently associated with the presence of mycobacterial disease with this model (Table 4).

**Table 3:** Comparison of selected variables between patients with and without evidence of mycobacterium infection

***Age, BMI, Exacerbations/Year and Pre-Operative Lung Function of patients without mycobacterium evidence compared to those with mycobacterium evidence (Mean ± SD)***			
	**No Mycobacterium**	**Mycobacterium**	**P value 95% CI**
No. of patients	112	14	
Male: Female ratio	89:23	9:5	0.303
Age, years	57.5 ± 10.39	63.0 ± 6.02	0.054
			(95% CI: −0.096-11.15)
BMI	23.9 ± 4.04	22.1 ± 4.36	0.110
			(95% CI: −4.15-0.42)
**Exacerbations/Year**	2.2 ± 2.40	5.3 ± 2.69	**<0.001**
			(Mann–Whitney Test, 95% CI not possible)
**FEV**_**1**_**, % predicted**	37.7 ± 17.17	25.0 ± 6.58	**<0.001**
			(95% CI: −17.55 -7.84)
FVC, % predicted	81.7 ± 21.44	74.1 ± 15.00	0.207
			(95% CI: −19.23-4.20)
**TLCO, % predicted**	42.6 ± 17.88	30.7 ± 9.11	**<0.001**
			(95% CI: −18.05-- 5.85)
RV, % predicted	218.1 ± 59.20	215.6 ± 63.17	0.881
			(95% CI: −36.11-31.02)
TLC, % predicted	127.8 ± 21.49	130.0 ± 23.10	0.722
			(95% CI: −9.99- 14.39)

Data shown as mean ± SD. FEV_1_: Forced expiratory volume in 1 sec; FVC: Forced vital capacity; TLCO: Transfer factor of the lung for carbon monoxide; RV: Residual volume; TLC: Total lung capacity. These variables are all shown as percentage predicted.

**Table 4:** Multivariate comparison of selected variables between patients with and without evidence of mycobacterium infection

	**Odds ratio (95% confidence interval)**	**P-Value**
Sex		
Male	0.79 (0.12, 4.98)	0.798
Female		
Age		
<60	0.76 (0.15, 3.78)	0.740
≥60 years		
BMI		
<18.5 kgm^−2^	0.13 (0.01, 1.50)	0.102
≥18.5 kgm^−2^		
**Exacerbation rate**		
<2 per year	50.24 (4.19, 602.01)	**0.002**
≥2 per year		
Pre-operation FEV1 (% predicted)		
<40%	1.08 (0.8, 15.45)	0.954
≥40%		
Pre-operation FVC (% predicted)		
<80%	0.24 (0.04, 1.37)	0.108
≥80%		
Pre-operation TICO (% predicted)		
<40%	0.17 (0.03, 1.09)	0.061
≥40%		
Pre-operation TLC (% predicted)		
<125%	5.86 (0.53, 64.6)	0.149
≥125%		
Pre-operation RV (% predicted)		
<200%	0.29 (0.03, 2.52)	0.261
≥200%		

Results of binary logistic regression. The odds ratio is the odds of mycobacterium (present) if risk factor category present relative to other risk factors. All odds ratios are adjusted for all other risk factors in the model.

## Discussion

This is the first study, to our knowledge, using resected lung specimens from LVRS/bullectomy procedures to estimate the prevalence of concurrent clinically unexpected mycobacterial infection in patients with severe COPD. 11% of 126 patients (10% of 142 specimens) had histological evidence of mycobacterial infection (necrotising granuloma, with or without AFB). A small number of previous studies [8,12,13] have likewise found evidence of a wide range of histopathological diagnoses in addition to emphysema in surgical specimens, including neoplastic nodules and interstitial fibrosis, however none of these studies have previously reported any evidence of mycobacterial disease on histology.

COPD and other chronic respiratory conditions have been shown to confer a high risk of NTM infection. One study [14], showed that patients, already diagnosed with NTM (according to the ATS criteria [2]), were likely to also have COPD (odds ratio: 15.7). This study also showed there was longstanding COPD (i.e. they had COPD for more than 5 years before NTM diagnosis) in 42.8% of all NTM patients [14]. Furthermore, inhaled steroid usage and dosage conferred an additional risk for NTM after adjustment for oral steroid use and comorbidities [14]. A similar US study [15] looked at diagnosed cases of NTM and found a high prevalence of co-morbid COPD in patients with NTM infection (28%). This present study differs by taking the opposite approach and providing an assessment of the prevalence of mycobacterial disease in a consecutive series of patients undergoing surgery for COPD, and importantly with no prior clinical indicators or suspicion of mycobacterial disease.

It is difficult to determine the clinical significance of these findings. These patients would not fulfil the criteria for NTM pulmonary disease and there was no morbidity that could be specifically attributed to these findings. In addition, outcome is difficult to determine following surgical removal of the areas that are likely to represent mycobacterial disease. In this study, the pre-operative FEV_1_ and TLCO were significantly lower, and the exacerbation rate (no. of COPD exacerbations in the year prior to surgery) was significantly higher for patients with evidence of mycobacterial infection compared to those patients without evidence of mycobacterial infection. However, it is not clear from our results whether these may be caused by the concurrent NTM infection in patients with COPD, or whether patients with more severe COPD (signalled by worse lung function and exacerbation rates) are more susceptible to NTM infection. The association was strongest on multivariate analysis between exacerbation rate and histological evidence of mycobacterial disease, however, it would not be sensible to exclude the importance of the variables found to be significant on the univariate analysis, because of the sample size.

There are other important messages that come out of these findings. Subclinical mycobacterial disease can be present in patients with severe COPD and this may have implications for COPD management. Long term, prophylactic macrolide therapy has recently been suggested as a therapeutic strategy for COPD patients with regular infections [16]. Before commencing this however, it is important to screen for NTM disease as macrolide monotherapy is contraindicated in such instances, due to the risk of macrolide resistance and worse prognosis [2]. These results indicate a further level of complexity to therapeutic decision-making, if there is a need to also exclude subclinical NTM infection as discovered in a significant proportion of surgical specimens.

Due to the nature of this study, there are several limitations. The diagnosis of mycobacterial disease in this study is based on histological rather than microbiological findings. There was no clinical suspicion of mycobacterial disease in these patients undergoing LVRS. As such, samples were routinely sent for histology but not for culture. This apparent limitation actually serves to highlight the importance and relevance of this work. There have been no previous similar studies suggesting such a high potential rate of mycobacterial disease. It demonstrates that samples should be sent for culture but also that mycobacterial disease needs to be actively considered in this population. The lack of definitive culture is a common occurrence in mycobacterial disease as highlighted by the lack of a positive TB culture in over 40% of treated TB cases in the UK [17]. The diagnosis of mycobacterial disease was nevertheless reasonably confident, with necrotising granulomas in all 14 patients and positive AFB staining in 8/14 specimens. Also, histological examination of the tissue has an advantage over microbiological diagnosis of being able to assess the activity and chronicity of the infection, through an assessment of the relative abundance of inflammation, fibrosis and necrosis. Certain fungi can cause necrotising granulomas such as histoplasmosis, although we would have expected to see these with a Grocott stain. It is likely that this study will tend to underestimate the number of patients with mycobacterial infection. Firstly, a proportion of the non-necrotising granulomas that were also found in a further 16 patients may represent burnt-out or inactive mycobacterial disease. However, on reviewing those without necrosis, no evidence of organisms was seen and our data suggest that this histological feature may therefore be an important one in assessing the risk of active disease. In addition, the non-necrotising (n = 15) group had clinical features more in common with the ‘non mycobacterial’ group and on a separate analysis had significantly different exacerbation rates, FEV1 and TLCO to the necrotising (n = 14) group) (data not shown). Secondly, this study would not capture patients with evidence of mycobacterial disease outside of the anatomical area biopsied. Although accurate differentiation between NTM and Mycobacterium tuberculosis is not possible without culture, TB was not suspected in any of the cases clinically and Interferon Gamma Release Assays (IGRA), although not perfect, were only positive in 1/5 patients where this was performed (this patient went on to culture M. kansasii which is known to produce a positive IGRA). It is also important to note that these results relate to COPD patients who had disease that was suitable for LVRS and may not necessarily be able to be generalised across all severe COPD patients.

In addition to evidence of mycobacterial infection, a wide range of other histological diagnoses were made in addition to emphysema. This has been seen in other similar studies [8,12] and is an indication of the pathology that co-exists with severe emphysema.

## Conclusion

A high proportion of patients with end-stage COPD requiring surgical treatment had histopathological evidence of clinically and radiologically unsuspected mycobacterial infection. Despite the study limitations of lack of culture data and the unknown clinical significance, this does provide an important clinical message. Mycobacteria are more common in COPD patients than suspected, particularly in patients with more severe disease or multiple exacerbations, and this should be considered and appropriate cultures obtained.

## Abbreviations

FEV_1_: Forced expiratory volume in 1 sec; FVC: Forced vital capacity; TLCO: Transfer factor of the lung for carbon monoxide; TLC: Total lung capacity; RV: Residual volume; LVRS: Lung volume reduction surgery; CF: Cystic fibrosis; COPD: Chronic obstructive pulmonary disease.

## Competing interest

The authors declare that they have no competing interests.

## Author’ contributions

AC analysed the data and drafted the manuscript. NH collected and analysed the data. DH reviewed the radiology. AN reviewed the histology. EC reviewed the histology. SC collected the data. PS performed the statistical analysis. RW analysed the data. SJ collected and analysed the data. ML conceived the study, analysed the data and drafted the manuscript. All authors read and approved the manuscript.

## Pre-publication history

The pre-publication history for this paper can be accessed here:

http://www.biomedcentral.com/1471-2466/14/124/prepub
